# Silent Sinus Syndrome of the Frontal Sinus: A Case Report

**DOI:** 10.7759/cureus.75516

**Published:** 2024-12-11

**Authors:** Maryam Khatoon, George McNally, Samit Ghosh

**Affiliations:** 1 Otolaryngology, Fairfield General Hospital, Northern Care Alliance NHS Foundation Trust, Manchester, GBR

**Keywords:** enophthalmos, facial asymmetry, frontal sinus, rhinoplasty, silent sinus syndrome

## Abstract

Silent sinus syndrome is a rare condition that typically affects the maxillary sinus, with only a few reported cases of frontal sinus involvement. Blockage of the sinus ostium leads to persistent hypoventilation, creating negative pressure and eventual sinus collapse. This report describes a previously undocumented case of facial asymmetry due to frontal silent sinus syndrome, following multiple childhood nasal injuries. A 23-year-old male presented with unilateral nasal obstruction, right-sided septal deviation and facial asymmetry. He sustained multiple childhood nasal injuries, requiring manipulation under anaesthetic of his nasal bones at four years old. Imaging reported symmetrical maxillary sinus volumes, with an opacified and collapsed left frontal sinus caused by obstruction of the sinus ostium, secondary to significant left-sided deviation of his bony nasal septum. Open septorhinoplasty was performed, with post-operative resolution of his cosmetic and functional nasal deformities. This report describes a case of frontal silent sinus syndrome causing ipsilateral hyperglobus and enophthalmos, successfully treated with open septorhinoplasty. Silent sinus syndrome rarely affects the frontal sinus and should be considered in asymptomatic patients with facial asymmetry. This advanced presentation highlights the importance of early recognition to prevent visual disturbance and proposes a perspective on disease progression.

## Introduction

Silent sinus syndrome is a rare condition characterised by unilateral chronic maxillary sinus atelectasis in the absence of rhinological symptoms [1]. The pathophysiology is thought to be due to a narrowed or blocked sinus drainage pathway, leading to persistent hypoventilation of the sinus cavity [2]. This creates a negative pressure, drawing in the sinus cavity walls, resulting in chronic opacification with atelectasis and eventual sinus wall collapse [2]. This presents clinically as progressive enophthalmos and hypoglobus, which, if left untreated, may lead to significant visual disturbance and orbital complications.

Silent sinus syndrome affecting the frontal sinus is exceptionally rare. A recent review article found a total of 147 published cases of silent sinus syndrome, with only one involving the frontal sinus [3,4]. In 2022, a second case of frontal silent sinus syndrome was published, reporting effective management with endoscopic sinus surgery [5]. This is the favoured treatment option to restore patency to the obstructed sinus ostium and prevent worsening facial asymmetry. This report describes a previously undocumented case of left frontal silent sinus syndrome, secondary to significant nasal deformity as a child, managed successfully with open septorhinoplasty.

## Case presentation

A 23-year-old presented with unilateral right-sided nasal obstruction and deviation. He did not report any other rhinological symptoms or complaints of facial pain. He had no past medical history, except for multiple nasal injuries as a child, requiring manipulation of his nasal bones under general anaesthesia at four years old.

**Figure 1:**
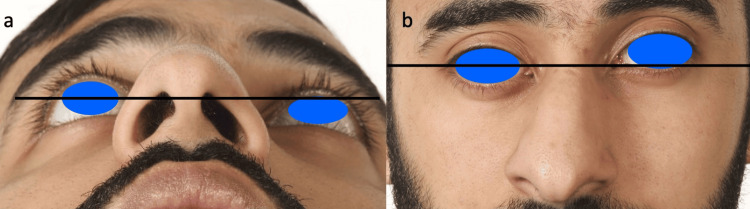
Clinical photography demonstrating right sided deviation of the nasal dorsum with annotation to illustrate left sided enophthalmos (a) and hyperglobus (b) secondary to silent sinus syndrome of the left frontal sinus. The patient consented and a written and signed consent statement was provided to the journal.

**Figure 2:**
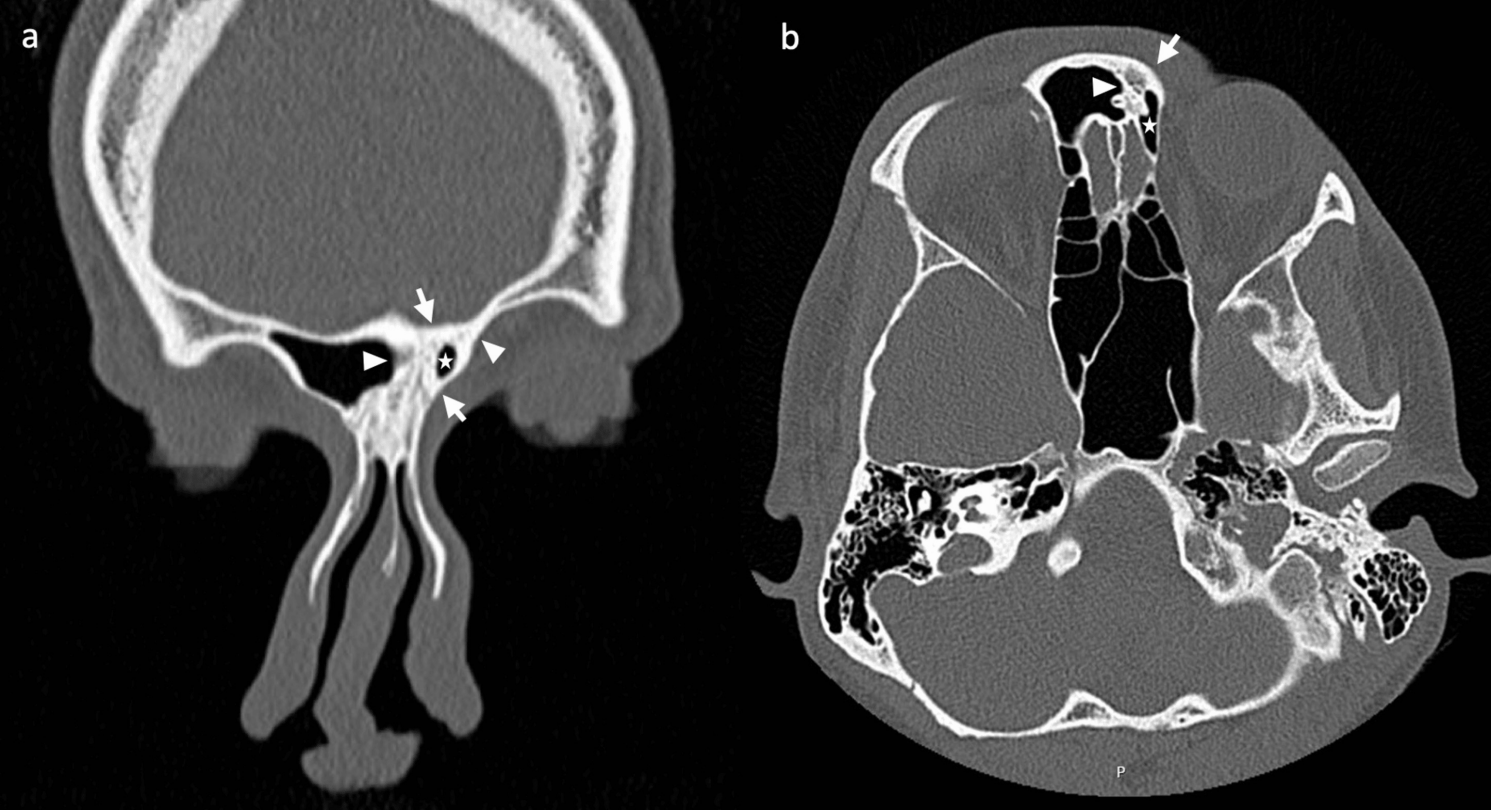
Coronal (a) and axial (b) slices of computed tomography scans demonstrating reduced left frontal sinus volume (star) secondary to retraction of the inter-frontal sinus septum to the left (arrowhead) and upwards displacement of floor of the left frontal sinus (arrowhead) with evidence of hyperostosis of the frontal bone (arrows).

On examination, there was a right-sided deviation of his nasal dorsum and marked facial asymmetry (Figure 1). The right eye appeared to be significantly lower and sunken compared to the contralateral side. His visual acuity and cranial nerve examination were both normal. Flexible nasendoscopy revealed a significantly deviated anterior nasal septum to the right. A computed tomography (CT) scan was requested, with a proposed diagnosis of silent sinus syndrome affecting the maxillary sinus. On the contrary, the CT reported symmetrical maxillary sinus volumes, with a partially opacified and collapsed left frontal sinus causing enophthalmos and upward displacement of the globe. The inter-frontal sinus septum and floor of the frontal sinus were both retracted inwards, with evidence of hyperostosis of the frontal bone (Figure 2). The quadrilateral cartilage was deviated to the right inferiorly, and the superior bony septum was significantly deviated to the left, narrowing the frontal sinus ostium. An open septorhinoplasty was performed to correct the bony and cartilaginous septal deformities, concurrently opening the occluded frontal sinus drainage pathway. At four months post-operatively, he reported resolution of his cosmetic and functional nasal deformities, with no further progression of his facial asymmetry.

## Discussion

Silent sinus syndrome is classically described as unilateral maxillary sinus opacification with orbital floor collapse in an otherwise asymptomatic patient [4]. Obstruction of the sinus ostium results in persistent hypoventilation, accumulation of secretions and subsequent gas reabsorption, causing negative pressure within the cavity. The resulting vacuum leads to inwards retraction of the sinus walls, with chronic opacification, atelectasis and eventual sinus collapse. This causes the classical facial appearance of ipsilateral enophthalmos and hypoglobus when the maxillary sinus is affected [2].

There are very few extra-maxillary presentations of silent sinus syndrome, with just three cases of ethmoid sinus involvement causing medial displacement of the globe, and only two reports of frontal sinus involvement: the first case presenting with enophthalmos and hyperglobus, and the second case presenting with enophthalmos alone [3,5-8]. On imaging, both patients had evidence of a large supra-agger frontal cell, otherwise known as a type III Kuhn cell, causing obstruction of the frontal sinus ostium. Tham et al. also reported a lateralised middle turbinate [5]. The clinical presentations of these cases share similarities with this case report in that they all observed a degree of ipsilateral enophthalmos, with or without hyperglobus, in the absence of sinusitis symptoms.

More recently, a small case series described frontal silent sinus syndrome in three patients with ipsilateral retraction of the inter-frontal sinus septum, as opposed to retraction of the frontal sinus floor [9]. However, these patients all presented with symptoms of chronic rhinosinusitis, including facial pain and did not report any evidence of facial symmetry. This is contradictory to the recognised definition of silent sinus syndrome, characterised by facial asymmetry with an absence of sinus symptoms [1]. These cases seem to fit more with a frontal variant of chronic maxillary atelectasis (CMA), defined as a persistent and progressive decrease in sinus volume associated with sinusitis symptoms and facial pain [10]. However, de Dorlodot et al. propose that CMA and silent sinus syndrome are part of the same disease spectrum, with the most severe cases of CMA presenting with facial asymmetry, with or without symptoms of sinusitis [11].

However, the case series did highlight that atelectasis leads to osteopenia, causing weakness and thinning of the sinus walls [9]. On review of the currently published cases of frontal silent sinus syndrome, it may be that the direction of orbital displacement depends on which cavity wall is most vulnerable to osteopenia at baseline. If the inter-frontal sinus septum is weakest, there may be no resultant facial asymmetry, whereas if the sinus floor is most vulnerable, this will lead to significant upward orbital displacement. Milder cases may develop enophthalmos alone, whereas more advanced cases, such as in this case report, may present with concurrent enophthalmos, hyperglobus and ipsilateral deviation of the inter-frontal sinus septum.

The primary goal of management is to relieve sinus obstruction and restore ventilation to prevent worsening facial asymmetry and visual disturbance. Endoscopic sinus surgery is the favoured treatment for maxillary silent sinus syndrome and has been successful for reported cases of frontal silent sinus syndrome [3,5]. While Naik et al. and Tham et al. reported CT findings of obstructing supra-agger cells, radiological imaging, in this case, indicated significant deviation of the nasal bones, causing obstruction of the frontal recess. This article suggests that the patient’s childhood nasal deformity, exaggerated by nasal growth and development during adolescence, was the pathogenesis for his frontal silent sinus syndrome. Realignment of the osseous and cartilaginous nasal septum via open septorhinoplasty successfully restored patency to his frontal sinus ostium and avoided the inherent risks associated with endoscopic sinus surgery, such as orbital injury and cerebrospinal fluid leak.

## Conclusions

This case reinforces that silent sinus syndrome is not a process limited to the maxillary sinus; thus, in asymptomatic patients presenting with facial asymmetry, silent sinus syndrome affecting the frontal sinus should be considered as a differential. It has highlighted that, in certain anatomical configurations, open septorhinoplasty can produce positive outcomes, whilst avoiding exposure to orbital or intracranial complications associated with endoscopic techniques. In conclusion, this unique case furthers our understanding of frontal silent sinus syndrome, highlights the importance of early recognition and provides a perspective on disease progression.
